# An Assessment of the Safety, Efficacy, and Tolerability of a Novel Scalp Treatment Regimen Combining a Hydroxy Acid-Based Scrub and Copper Tripeptide Serum in the Management of Seborrheic Dermatitis in Adults

**DOI:** 10.7759/cureus.70108

**Published:** 2024-09-24

**Authors:** Maheshvari N Patel, Nayan Patel, Apeksha Merja, Saurav Patnaik

**Affiliations:** 1 Clinical Research, NovoBliss Research Private Limited, Ahmedabad, IND; 2 Pharmacology, Swaminarayan University, Ahmedabad, IND; 3 Dermatology, NovoBliss Research Private Limited, Ahmedabad, IND; 4 Cosmetology, Anveya Living Private Limited, Gurgaon, IND

**Keywords:** adherent scalp flaking score, copper peptide, dandruff, dry scalp, seborrheic dermatitis

## Abstract

Background and objective

Scalp dandruff, a prevalent dermatological condition marked by flaking and itching, affects a large segment of the population. Sun exposure, genetics, and dryness, along with winter conditions, indoor heating, and hard water, all contribute to moisture loss and scalp imbalance. This study aimed to evaluate the safety, efficacy, and tolerability of a novel regimen combining a scalp scrub with hydroxy acid derivatives for exfoliation and dandruff removal, and a serum containing copper tripeptide-1, witch hazel (*Hamamelis virginiana)*, hesperidin, hyaluronic acid, and pea protein (*Pisum sativum*) for hydration and scalp health.

Methods

This prospective, interventional, open-label, single-center study assessed the safety, efficacy, and in-use tolerability of a scalp scrub and serum regimen for mild to moderate dandruff. Ethical approval was granted by ACEAS - Independent Ethics Committee and all participants provided written consent. The study evaluated changes in Adherent Scalp Flaking Score (ASFS), scalp hydration with the Corneometer^®^ CM 825, hair thickness, hair density, and scalp condition using CASLite Nova. Scalp appearance and treatment usage perception were evaluated on day 1, day 8, and day 15. Statistical analysis was conducted using SPSS Statistics v29.0.1.0 (IBM Corp., Armonk, NY) and Microsoft Excel 2019, with results reported as p-values and 95% confidence intervals (CI).

Results

As per the ASFS scale for adherent scalp flaking, a significant improvement of 61.24% (p<0.0001) was observed by day 15. Assessments with CASLite Nova showed significant increases in hair density and hair thickness by 12.48% (p<0.0001) and 25.36% (p<0.0001), respectively. Regarding scalp condition, the proportion of individuals with a dry scalp and significant keratin decreased from 65.52% at baseline to 6.9% by day 15. The incidence of dry scalp with fine dandruff also decreased from 6.9% at baseline to 0% by day 15. Conversely, the percentage of individuals with a dry scalp and some keratin increased from 27.59% at baseline to 48.28% by day 15. Also, the proportion of subjects with a normal scalp, characterized by good hair density and thickness, increased from 0% at baseline to 44.83% by day 15. Scalp skin hydration, measured by Corneometer® CM 825, showed a significant improvement of 76.24% (p<0.0001) from baseline to day 15. Overall, there were notable enhancements in scalp appearance, with reductions in itchiness, redness, roughness, and scaliness. All subjects reported satisfaction with the treatment.

Conclusions

The ThriveCo scalp regimen (scalp scrub + serum) has been demonstrated to be both effective and safe in treating and reducing scalp dandruff in healthy adults. The regimen significantly reduced ASFS by effectively removing visible scalp flakes from day 1 to day 15 with continuous use. Furthermore, the kit improved overall scalp condition, increased skin hydration, and enhanced the scalp's appearance. The components include scalp scrub with hydroxy acid derivatives for exfoliation and dandruff removal, and a serum containing copper tripeptide-1, *Hamamelis virginiana*, hesperidin, hyaluronic acid, and pea protein (*Pisum sativum*) for hydration and overall scalp health over the 15-day treatment period. The synergistic effects of this regimen work to clear visible dandruff flakes, reduce adherent scalp flaking, rejuvenate the scalp, and simultaneously strengthen the hair.

## Introduction

Dandruff, or pityriasis capitis (seborrheic dermatitis of the scalp), tends to persist despite a variety of treatments [1]. It is characterized by increased scalp scaling, aggravated by factors such as sun exposure, genetic predispositions affecting the epidermal barrier, and dryness [2-4]. Winter conditions often exacerbate dandruff due to low relative humidity, while indoor heating systems further deplete moisture and increase air pollution. Persistent dryness leads to the buildup of dead skin cells, contributing to dandruff [5]. Additionally, hard water, which contains high levels of calcium and magnesium, can leave mineral deposits on the scalp and hair, disrupting the scalp's moisture balance and leading to dryness, irritation, and flakiness that can also contribute to dandruff [6]. Dandruff typically occurs in three key stages: infancy (the first three months), puberty, and adulthood, with peak prevalence between the ages of 40 and 60 years. Affecting around 50% of adults globally, it is more common in males than females [7].

Primarily, there are two types of dandruff: adherent and non-adherent. Adherent dandruff refers to flakes of dandruff that stick closely to the scalp. Non-adherent dandruff refers to dandruff flakes that are less sticky or less attached to the scalp compared to adherent dandruff [8]. Methods such as the Adherent Scalp Flaking Score (ASFS), Corneometer® CM 825, and CASLite Nova have been introduced to enhance accuracy and detailing in dermatological evaluations. The ASFS involves dividing the scalp into eight zones and categorizing the severity of flaking as mild, moderate, or severe [9,10]. Corneometer® CM 825 is used to measure scalp hydration levels [11].

Current solutions for scalp dandruff often fall short in terms of providing lasting relief and comprehensive care. The new scalp regimen we discuss addresses this issue with an innovative approach, featuring a scrub and serum. The scrub contains a blend of alpha-hydroxy acid (AHA), beta-hydroxy acid (BHA), polyhydroxy acid (PHA), salicylic acid, glycolic acid, and orange peel oil, working together to target dandruff at its source. Complementing this, the serum is formulated with copper tripeptide-1, *Hamamelis virginiana*, hesperidin, hyaluronic acid, and pea protein (*Pisum sativum*). This powerful combination promotes a healthier scalp, reduces flaking, and helps prevent dandruff recurrence.

This study aimed to evaluate the safety, effectiveness, and tolerability of a scalp regimen designed to treat dandruff. The regimen included a scalp scrub containing hydroxy acid derivatives and a serum with copper tripeptide-1, *Hamamelis virginiana*, hesperidin, hyaluronic acid, and pea protein (*Pisum sativum*). Conducted as an open-label, single-arm, single-center, prospective interventional clinical trial, the research focused on assessing changes in flaking scores, skin hydration, hair thickness, hair density, and overall scalp condition.

## Materials and methods

Ethical conduct of the study

This study was conducted per the principles outlined in the New Drugs and Clinical Trials Rules 2019, ICH Guidance E6 (R2) on 'Good Clinical Practice,' ICMR's National Ethical Guidelines for Biomedical and Health Research Involving Human Participants, 2017, and the Declaration of Helsinki (Brazil, October 2013). Ethical approval for the study plan was obtained from the Ethics Committee before the commencement of any study-related activities. All participants provided signed informed consent before enrolment in the study. The consent process included a detailed explanation of the study objectives, procedures, confidentiality measures, and the voluntary nature of participation.

Furthermore, this clinical study was registered with the Clinical Trial Registry of India (CTRI) [CTRI/2023/11/060289]. This comprehensive ethical framework ensures the study's compliance with international and national ethical standards, safeguarding the rights, safety, and well-being of all participating subjects.

Study design

This was a prospective, interventional, open-label, single-center, single-arm, clinical study aimed to assess the safety, effectiveness, and tolerability of the test product from January 1, 2024, to January 30, 2024, involving a total of 32 adult subjects aged 18 to 55 years with mild to moderate scalp dandruff. The study duration was 15 days. The primary objective was to evaluate changes in scalp dandruff using ASFS, scalp phototrichogram using CASLite Nova, and scalp hydration using Corneometer® CM 825. The secondary objective was to evaluate changes in the general appearance of the scalp and assess the subject’s perception of the test treatment.

Subjects were screened based on predefined inclusion and exclusion criteria to ensure a homogeneous study population. The inclusion criteria required participants to be healthy adults aged 18-50 years (both inclusive), with good general health confirmed by recent medical history. Both healthy males and non-pregnant, non-lactating females were eligible. Females of childbearing potential had to furnish a self-reported negative urine pregnancy test and to be using hormonal contraception for at least six months, agreeing to continue its use throughout the study. Participants needed to have mild to moderate dandruff, as indicated by ASFS, and have left their scalp unwashed and untreated for five days. Additionally, participants needed to be willing and able to follow study directions, attend all specified visits, and provide written informed consent.

Exclusion criteria included individuals with known allergies or sensitivities to the test treatment ingredients, a history of dermatological conditions of the scalp other than dandruff, or a history of alcohol or drug addiction. Subjects using other dandruff control products or hair growth treatments during the study were excluded, as were those planning to shave their scalp hair. Individuals with skin irritation, open wounds, cuts, or any facial dermatological conditions that could interfere with assessments were also excluded. Additionally, those on medications affecting scalp response, with concurrent skin diseases, or who had taken systemic corticosteroids, anti-bacterial drugs, immunosuppressants, or undergone abrasive facial procedures in the past 30 days were excluded. Subjects who were likely to engage in excessive sun exposure or who were pregnant, breastfeeding, or planning to become pregnant during the study period were also excluded.

Study procedure

The study was conducted over three visits with a total treatment duration of 15 days. Visit 1 (day 1) encompassed screening, enrolment, and initial assessments pre- and post-hair wash after usage of test treatment at 60 minutes. Visit 2 (day 8) involved intermediate parameter evaluations, while visit 3 (day 15) included final parameter assessments and the end of the study. Table 1 provides the details about the test regimen.

**Table 1:** Details of the test regimen Note: ThriveCo Hair Vitalizing Rosemary Shampoo was provided along with the test treatment to ensure consistency and avoid deviations from the instructions among all patients

ThriveCo Scalp Kit		
Name	ThriveCo Exfoliating Scrub	ThriveCo Vitalizing Serum
Mode of usage	Apply the scrub on the dry scalp and massage it into the hair roots. Leave it on for 10 minutes before rinsing thoroughly	Section hair and apply evenly across the scalp using the dropper. Massage in with fingertips. Use on damp or dry hair. Do not rinse out
Active ingredients	Alpha-hydroxy acid (AHA), beta-hydroxy acid (BHA), polyhydroxy acid (PHA), salicylic acid, glycolic acid and orange peel oil	Copper tripeptide-1, *Hamamelis virginiana *(witch hazel), hesperidin, hyaluronic acid, and pea protein (*Pisum sativum*)
Frequency	2 times a week	Daily
Route of administration	Topical	
Manufacturer	Anveya Living Private Limited	

**Figure 1:**
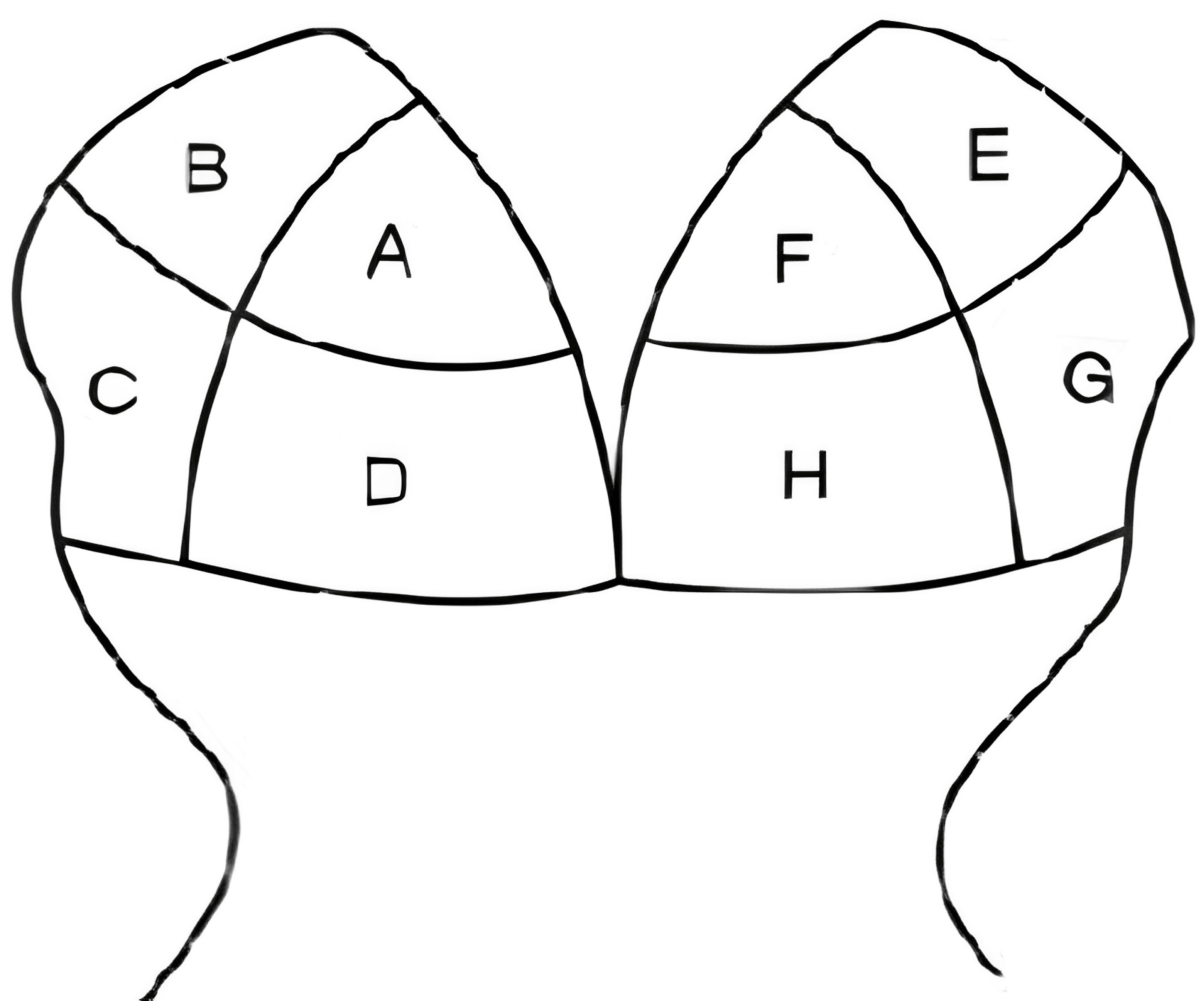
Division of scalp into eight sections for ASFS grading ASFS: Adherent Scalp Flaking Score

ASFS was evaluated by dividing each subject’s scalp into eight zones, and each section of the scalp was assessed for the presence of dandruff flakes adhering to the scalp skin using a 0-10 scale (0: no flakes; 10: heavy flakes). The final or total ASFS was determined by summing up the grades for all eight zones on the scalp, which provided a possible score between 0 and 80. The subject score was categorized as follows: 1: mild (16-24), 2: moderate (25-34), and 3: severe (35-80). The evaluation was performed before and after usage of the test treatment: T60 min on day 1, day 8, and day 15. Figure 1 presents the reference images related to ASFS.

**Figure 2:**
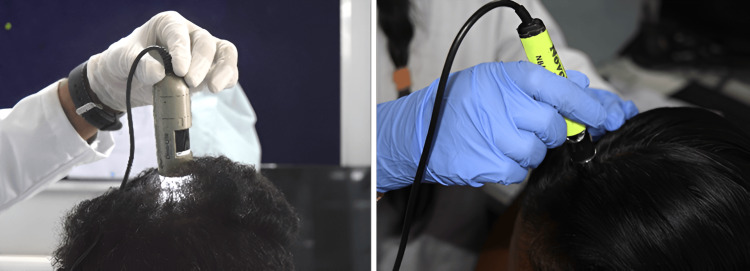
Phototrichogram using CASLite Nova (left); evaluating skin hydration using Corneometer® CM 825 (right)

Scalp phototrichogram is a non-invasive, reproducible method that is based on the manual marking of selected scalp areas on images taken at a close range from target areas on the scalp skin. The study staff had selected a small area, i.e., 1 x 1 cm (1 cm^2^) on the subject’s scalp 30 cm from the tip of the nose to the vertex - possibly the center of the vertex area. A permanent ink marker was used to standardize the location of the assessment and the same site was captured in-camera using the instrument. The subject’s scalp condition was assessed by instrumental evaluation, from a designated marked area on the scalp by CASLite Nova, as shown in Figure 2(a).

Hydration measurement was based on capacitance measurements of the stratum corneum, the uppermost layer of the skin, with dielectric properties changing with improved hydration. The Corneometer® CM 825 measured the change in the dielectric constant due to changes in skin surface hydration, altering the capacitance of a precision capacitor. Scalp skin hydration measurements were taken from the selected area on the subject’s scalp, as shown in Figure 2(b).

Statistical analysis

Continuous variables were described using descriptive statistics [N, mean, standard deviation (SD), median, and range]. Categorical variables were expressed using frequency and percentage along with graphical presentations whenever required. The statistical analysis was done using SPSS Statistics v29.0.1.0 (IBM Corp., Armonk, NY) and Microsoft® Excel 2019 software with a 5% level of significance. Subjects who withdrew from the study were not included in the statistical analysis.

Data handling and analysis

All data were carefully reviewed and refined before analysis to ensure accuracy and completeness. Frequency analyses and cross-tabulations were performed to ensure data accuracy and consistency. Missing data were addressed through appropriate imputation methods or excluded from the analysis, depending on the extent and nature of the missingness. The results of the statistical tests, including p-values, were reported with corresponding confidence intervals (CI) to provide a measure of precision and reliability.

Sample size determination

A sample size calculation was performed to ensure sufficient power to detect clinically meaningful differences. Based on this calculation, a total of 32 subjects were enrolled.

## Results

Demographic and other baseline characteristics

**Figure 3:**
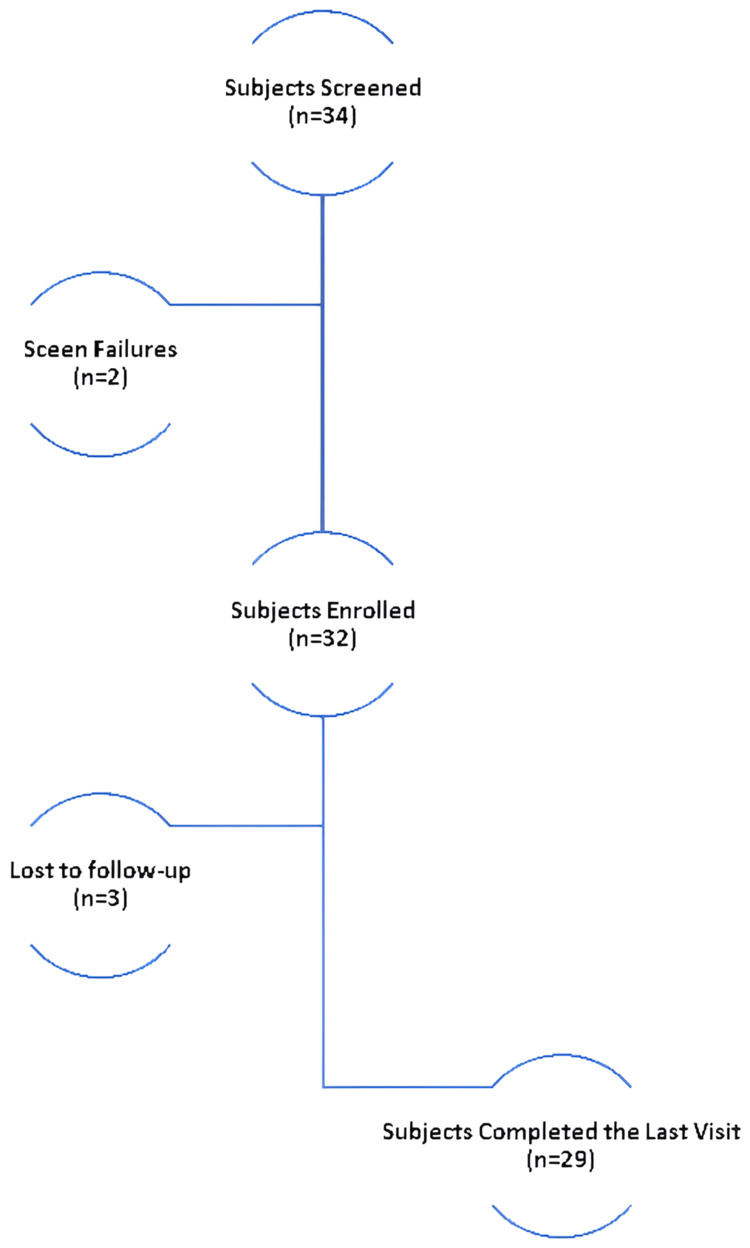
Subject characteristics

A total of 32 participants, aged between 18 and 50 years, were enrolled in the study, with 29 completing the protocol. Despite having three dropouts, comprehensive data for both pre- and post-intervention scalp assessments were successfully gathered, ensuring robust analytical outcomes. The study demonstrated high adherence to the intervention and assessment schedules. As shown in Figure 3, the cohort consisted of 53.13% females (n=17) and 46.88% males (n=15). The average age of participants was 27.16 ± 7.51 years, with a mean height of 157.56 ± 10.83 cm and an average weight of 59.53 ± 12.51 kg.

Primary efficacy endpoints

ASFS Scoring Scale

**Figure 4:**
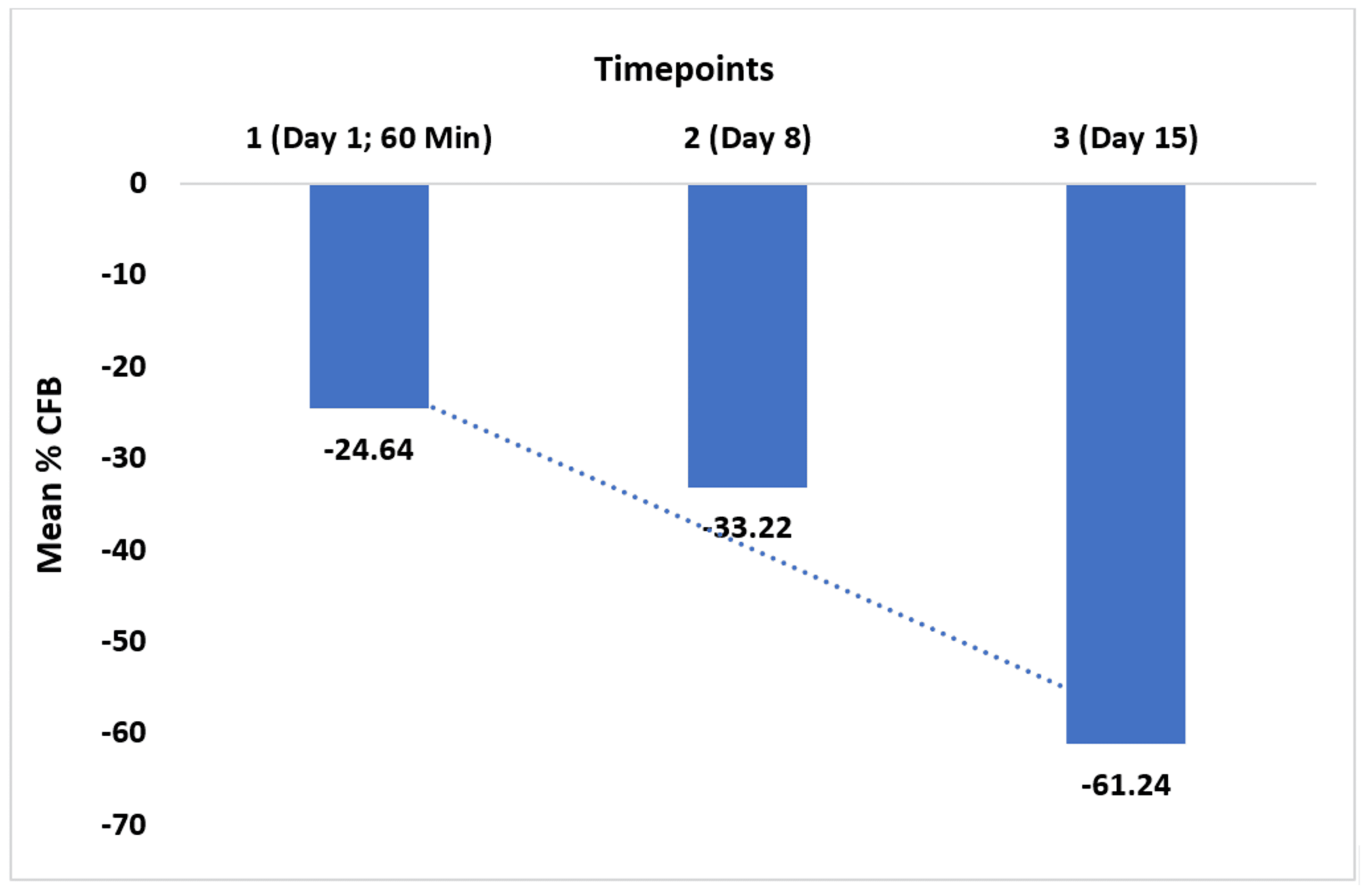
Percentage change in Adherent Scalp Flaking Score %CFB: percentage change from baseline

Initially, the baseline ASFS was 30.76. This decreased to 23.17 after the hair wash, representing a 24.64% reduction (p<0.001). After eight days, the ASFS significantly dropped further to 20.31, a 33.22% reduction (p<0.001). After 15 days, the ASFS decreased even more to 11.79, indicating a 61.24% reduction (p<0.001) (Figure 4).

On day 1, 6.90% of subjects had a mild score for adherent scalp flaking, while 93.10% had a moderate score. After applying the test product, 75.86% of subjects had a mild score and 24.14% had a moderate score. On day 8, 92.31% of subjects had a mild score and 7.69% had a moderate score. By the end of the study, 17.24% of subjects had a mild score, and 82.76% had no detectable flaking.

Hair Density, Hair Thickness, and Scalp Condition Based on CASLite Nova

**Figure 5:**
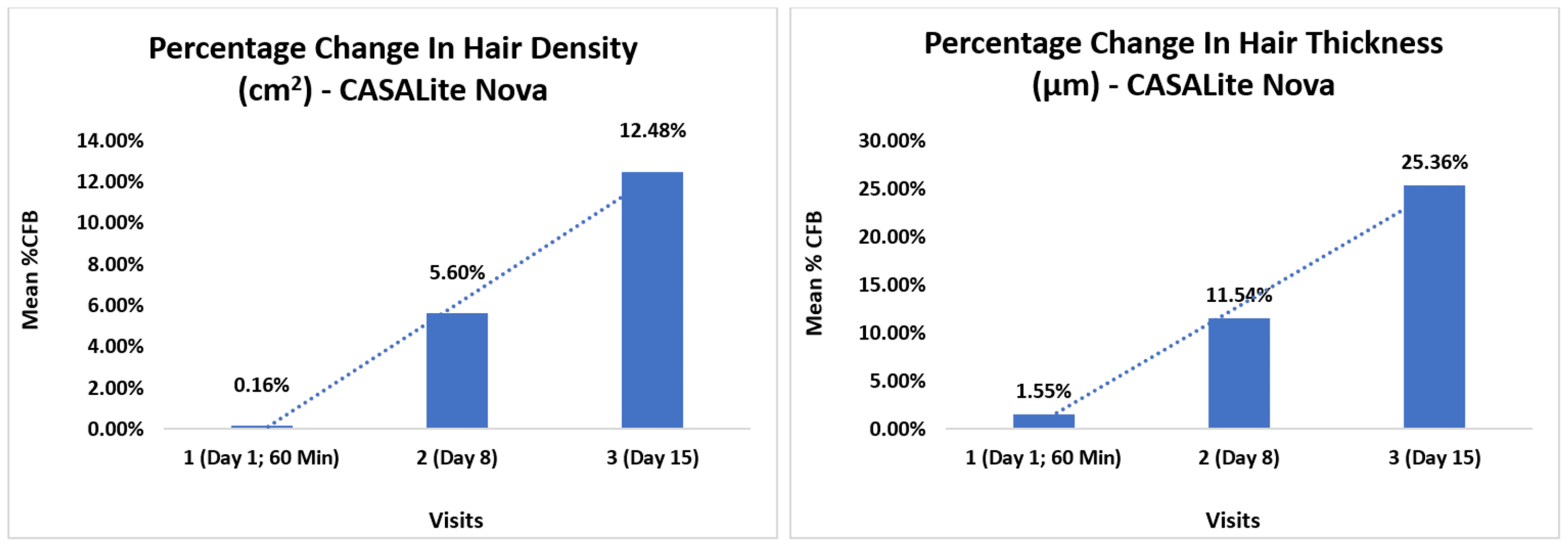
Percentage change of hair density and hair thickness using CASALite Nova %CFB: percentage change from baseline

Hair density measurements taken before treatment on day 1 and post-treatment at T60 minutes on day 1, day 8, and day 15 revealed mean values of 231.14 sqcm, 231.45 sqcm (p>0.05), 243.85 sqcm (p<0.0001), and 259.59 sqcm (p<0.0001), respectively. Additionally, CASLite Nova measurements for hair thickness demonstrated a statistically significant increase, with mean values of 13.66 µm, 13.86 µm (p>0.05), 15.15 µm (p<0.0001), and 17.00 µm (p<0.0001), at baseline, before treatment and at T60 minutes on day 1, day 8, and day 15 respectively. The percentage change in hair density and thickness is illustrated in Figure 5. 

**Figure 6:**
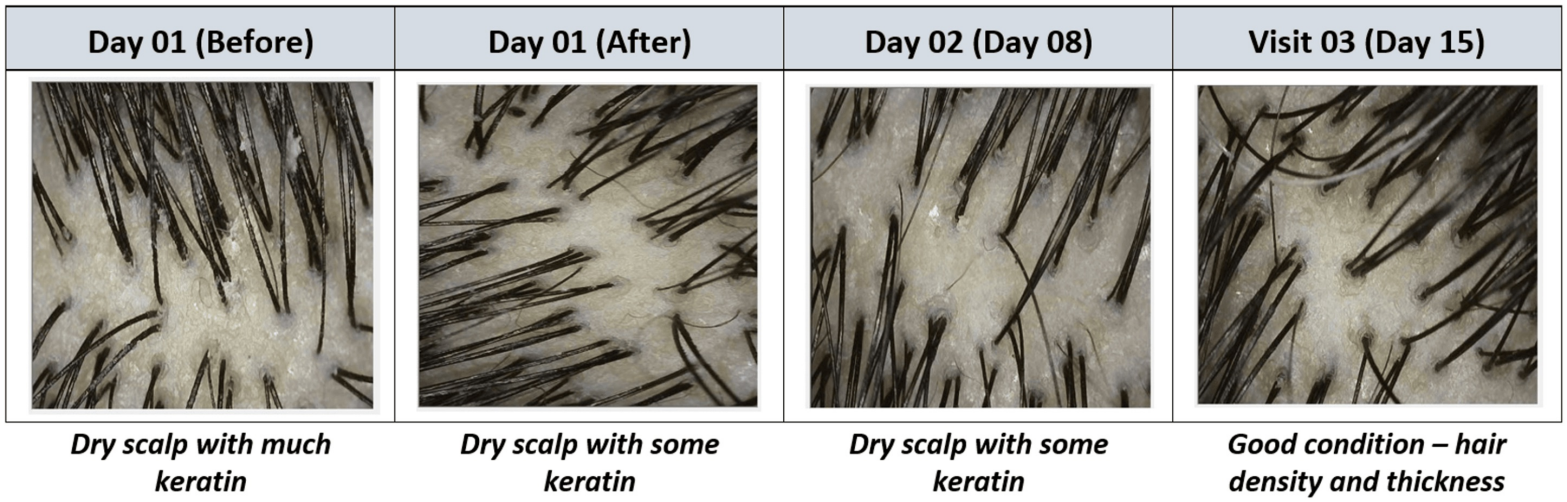
Phototrichograms of scalp condition assessed using CASLite Nova

Concurrently, scalp condition assessed using CASLite Nova demonstrated notable improvements post-study duration. Initially, 65.52% of participants had a dry scalp with much keratin. After 15 days of the study intervention, 44.83% of participants showed improvements, with their scalps classified as normal and in good condition. Further details are presented in Table 2 and Figure 6.

**Table 2:** Frequency and percentage of scalp condition

Scalp condition	Visit 1 (before)	Visit 1 (after)	Visit 2 (day 8)	Visit 3 (day 15)
	Count (%)			
Dry scalp with fine keratin	0 (0%)	0 (0%)	0 (0%)	0 (0%)
Dry scalp with much keratin	19 (65.52%)	5 (17.24%)	2 (7.69%)	2 (6.9%)
Dry scalp with fine dandruff	2 (6.9%)	3 (10.34%)	1 (3.85%)	0 (0%)
Dry scalp with some keratin	8 (27.59%)	21 (72.41%)	23 (88.46%)	14 (48.28%)
Normal scalp, good condition hair density and thickness	0 (0%)	0 (0%)	0 (0%)	13 (44.83%)

Scalp Skin Hydration Measured Using Corneometer® CM 825

Skin hydration was assessed using Corneometer® CM 825, revealing significant improvements. The mean changes in scalp hydration were 10.03, 13.77, 12.69, and 17.36 at baseline before product usage, at T60 minutes post-application, day 8, and day 15, respectively (p<0.001). These results demonstrate the test treatment's effectiveness in enhancing and maintaining optimal skin hydration levels.

Secondary efficacy endpoints

Change in Scalp Appearance: Itchiness, Redness, Roughness, and Scaliness

Itchiness: 62.07% of participants experienced mild scalp skin itchiness initially, with 34.48% reporting moderate itchiness, and 3.45% showing no signs of itchiness. Following 60 minutes of using the test treatment, 75.86% experienced mild itchiness, while 24.14% showed no signs of itchiness. By day 8, 50% of participants reported mild itchiness and an equal proportion displayed no signs of itchiness. On day 15, 6.90% experienced mild itchiness, while 93.10% exhibited no signs of skin itchiness.

Redness: 86.21% of subjects exhibited mild skin redness initially, while 3.45% displayed moderate redness, and 10.34% showed no signs of redness. After 60 minutes of using the test treatment, 55.17% experienced mild redness, while 44.83% showed no signs of redness. By day 8, 26.92% of subjects had mild redness, with 73.08% displaying no signs of redness. On day 15, 10.34% experienced mild redness, while 89.66% exhibited no signs of skin redness.

Roughness: 13.79% of subjects exhibited mild skin roughness initially, while 82.76% and 3.45% of subjects exhibited moderate and no sign of skin roughness. On day 1, before and post-60 min of the product usage, 82.76%, 13.79%, and 3.45% of the subjects had mild, moderate, and no sign of skin roughness respectively. On day 8, 69.23%, and 30.77% of subjects had mild and no sign of skin roughness. While on day 15, 17.24% and 82.76% of subjects had mild and no sign of skin roughness.

Scaliness: 89.66% of subjects exhibited mild skin scaliness, while 6.90% displayed moderate scaliness, and 3.45% showed no signs of scaliness. After 60 minutes of usage, 82.76% of subjects experienced mild scaliness, while 17.24% showed no signs of scaliness. On day 8, 30.77% of subjects exhibited mild scaliness, while 69.23% showed no signs of scaliness. By day 15, 6.90% experienced mild scaliness, while 93.10% exhibited no signs of skin scaliness.

Change in Subject Response Index Based on 9-Point Hedonic Scale

This 9-point hedonic scale features questions evaluating the test product’s effectiveness in several areas: reduction of scalp itchiness, redness, roughness, scaliness, and dryness; effectiveness in reducing dandruff for cleaner, healthier hair; combating dandruff effectively; strengthening hair; maintaining a clean and fresh feeling for the hair and scalp; providing softness and smoothness to the hair; keeping the scalp hydrated; and overall satisfaction. Respondents rate each aspect on a scale of 1 to 9, where 1 means "extremely dissatisfied," 2 means "very dissatisfied," 3 means "moderately dissatisfied," 4 means "slightly dissatisfied," 5 means "don't know," 6 means "slightly satisfied," 7 means "moderately satisfied," 8 means "very satisfied," and 9 means "extremely satisfied."

Among the total participants, 24.14% had used other products previously, while 75.86% had not used any products. The test treatment demonstrated significant improvements compared to earlier products. For reducing scalp itchiness, redness, roughness, scaliness, and dryness, 17.24% of subjects felt their previous product was effective, while 96.56% agreed that the test treatment achieved these benefits after 15 days. Regarding dandruff reduction for cleaner, healthier hair, only 15.63% found their earlier product effective, whereas 100% of subjects confirmed that the test treatment effectively reduced dandruff and provided a cleaner, healthier scalp.

The earlier product had limited success in fighting dandruff and strengthening hair, with only 15.63% reporting satisfaction, but the test treatment saw unanimous agreement (100%) in effectively combating dandruff and strengthening hair. Similarly, the earlier product was deemed effective by 12.5% for providing clean and fresh hair/scalp, while the test treatment was endorsed by 100% of subjects for delivering a clean and fresh feeling. Regarding softness and smoothness, only 12.5% were satisfied with their earlier product, compared to 100% satisfaction with the test treatment. For scalp hydration, 12.5% found the earlier product effective, while 100% agreed the test treatment provided adequate hydration. Overall, 18.76% were satisfied with their previous product, whereas 100% of subjects expressed satisfaction with the test treatment after 15 days.

## Discussion

Scalp issues like dandruff, flaking skin, and itching are common dermatological problems affecting people of all ages, and these often pose challenges in terms of effective treatment and patient satisfaction. This study aimed to assess the safety, efficacy, and tolerability of a new scalp regimen, which comprises a scalp scrub and serum, for treating mild to moderate dandruff in healthy adults. This was an open-label, single-arm, single-center clinical trial, that evaluated changes in ASFS before and after treatment, as well as modifications in hair thickness, hair density, overall scalp condition, and treatment perception.

AHAs have previously been reported to enhance the therapeutic benefits of topical corticosteroids in treating scalp conditions. AHAs are commonly used for skin exfoliation. When used in rinse-off products, AHAs quickly remove dead skin cells within minutes, making their application both extensive and efficient. BHAs, such as salicylic acid, act as keratolytic agents, effectively removing scaly, hyperkeratotic skin, thereby helping to break down and remove the outer layer of skin that becomes thickened or scaly. It works by disrupting the adhesion between corneocytes, the cells in the outermost layer of the skin, making it easier to shed dead skin cells. This action helps in treating conditions characterized by excessive skin buildup, such as dandruff and psoriasis. Due to its effectiveness in managing such conditions, salicylic acid is frequently included in various dermatological preparations and products designed for treating dry, flaky, or scaly skin. Its ability to exfoliate and improve the texture of the skin makes it a valuable ingredient in formulations aimed at promoting a healthier scalp and clearer skin [12,13].

Glycolic acid helps maintain moisture, condition, and hydrate the hair, while also fortifying it to minimize breakage. Additionally, it improves the hair's resilience to high temperatures from heat styling [14]. Peptide-copper complexes are specialized compounds used to stimulate hair growth. These complexes combine peptides, which are short chains of amino acids, with copper, a trace mineral essential for collagen and elastin production. They work by activating hair follicles, promoting their growth phase, and enhancing scalp circulation to deliver nutrients and oxygen. Typically, these complexes are incorporated into topical products like serums or creams, which are applied directly to the scalp. The formulations include safe diluents and carriers that aid in the effective delivery and absorption of the active ingredients, making them a targeted and non-invasive solution for encouraging hair growth [15].

Witch hazel (*Hamamelis virginiana*), a plant native to North America, was traditionally used by Native Americans for treating scalp conditions. Its anti-inflammatory and astringent properties make it effective for soothing irritation, reducing redness, and controlling excess oil. Witch hazel is still valued for these benefits and is commonly found in modern skincare products for its ability to tighten skin, and promote overall scalp health [16].

Hyaluronic acid (HA) functions as both a moisture-enhancing and conditioning agent. It is particularly beneficial for maintaining scalp hydration and health. Low-molecular-weight hyaluronic acid (LMW-HA) deeply penetrates the scalp, providing intensive moisture and supporting cellular regeneration, which can help alleviate dryness and irritation. High-molecular-weight hyaluronic acid (HMW-HA) forms a protective, viscoelastic film on the scalp, which reduces moisture loss by preventing water evaporation. This occlusive effect helps keep the scalp hydrated and balanced. Additionally, medium-molecular-weight hyaluronic acid (MMW-HA) shares similar hydrating and moisture-retaining properties, contributing to overall scalp hydration. Together, these forms of hyaluronic acid work to enhance scalp moisture levels, soothe dryness, and improve scalp health, creating an optimal environment for healthy hair growth [17,18].

Pea protein enhances hair growth by supplying essential amino acids and biotin, which are vital for hair strength and repair. Its high nutrient content supports healthy hair follicles and improves scalp hydration, reducing dryness and creating a favorable environment for hair growth. By strengthening hair strands and improving elasticity, pea protein helps prevent breakage and split ends, promoting longer, healthier hair. Additionally, its gentle, non-irritating nature makes it suitable for sensitive scalps, further contributing to its effectiveness in hair care formulations [19].

Hesperidin, a bioflavonoid in citrus fruits, offers several benefits for scalp health. It promotes wound healing, reduces inflammation, and provides UV protection, addressing issues like irritation and sun damage. Its antimicrobial properties combat infections and dandruff, while its skin-lightening effect can help with hyperpigmentation. Hesperidin also maintains the scalp's epidermal barrier, protects against oxidative stress, and inhibits MAPK pathways, contributing to overall scalp health. Affordable, widely available, and safe, hesperidin is a valuable option for managing various scalp conditions [20].

ASFS has been extensively validated as a reliable method for evaluating dandruff severity. A study by Patel et al. demonstrated that a significant decrease in ASFS indicates the effectiveness of hair washing in reducing adherent scalp flakes [21,22]. Similarly, the current study also shows a substantial improvement in ASFS. In the alopecia study, the scalp conditions observed at the last visit were as follows: 6.9% had a dry scalp with significant keratin, 34.48% had a dry scalp with some keratin, 55.17% had a normal scalp with good hair density and thickness, and 3.45% had a normal scalp in excellent condition [23]. In contrast, the current study found that 6.9% had a dry scalp with significant keratin, 48.28% had a dry scalp with some keratin, and 44.83% had a normal scalp with good hair density and thickness.

After using the test treatment, the findings indicate a substantial, statistically significant decrease in dandruff. Hair density improved by 1.12 times, hair thickness by 1.24 times, and skin hydration increased by 1.73 times. Most participants exhibited no signs of skin redness, itchiness, roughness, or scaliness after the treatment. This notable improvement underscores the efficacy of the test treatment in reducing scalp concerns and enhancing overall scalp health. Visible changes in scalp photographs from baseline to post-15-day usage showed reduced visible flakes, with the majority of subjects having a normal scalp and good hair thickness and density.

However, the study has several limitations that need to be acknowledged. Firstly, the relatively short treatment duration of 15 days may not capture long-term effects or variations in individual responses. This limited timeframe may not be sufficient to observe potential side effects or the sustainability of the treatment’s benefits over a longer period. Additionally, a larger sample size would provide more robust data and improve the reliability of the findings. Future research should address these limitations by conducting long-term studies with larger, more diverse populations and controlling for these variables to validate and expand upon the current findings.

## Conclusions

The ThriveCo Scalp Regimen, consisting of a scalp scrub and serum, has been clinically demonstrated to be both effective and safe in treating and reducing scalp dandruff in healthy adults. Over a 15-day treatment period, the regimen showed a significant reduction in ASFS, effectively removing visible scalp flakes from day 1. Additionally, the regimen improved overall scalp condition by enhancing skin hydration and rejuvenating the scalp's appearance. The scalp scrub, formulated with hydroxy acid derivatives, provides exfoliation and dandruff removal, while the serum, enriched with copper tripeptide-1, *Hamamelis virginiana*, hesperidin, hyaluronic acid, and pea protein (*Pisum sativum*), promotes hydration and scalp health. Together, these components work synergistically to clear visible dandruff, reduce adherent flaking, rejuvenate the scalp, and fortify the hair.
